# Tempo-Spatial Variation of Vegetation Coverage and Influencing Factors of Large-Scale Mining Areas in Eastern Inner Mongolia, China

**DOI:** 10.3390/ijerph17010047

**Published:** 2019-12-19

**Authors:** Aman Fang, Jihong Dong, Zhiguo Cao, Feng Zhang, Yongfeng Li

**Affiliations:** 1 School of Environment Science and Spatial Informatics, China University of Mining and Technology, Xuzhou 221116, China; fangaman123@163.com (A.F.); sxlyf01@163.com (Y.L.); 2 State Key Laboratory of Water Resource Protection and Utilization in Coal Mining, Beijing 100011, China; zgcao2008@163.com; 3 China Coal Technology & Engineering Group Tangshan Research Institute, Tangshan 063000, China; tszf2014@163.com

**Keywords:** grassland vegetation, coal mining, temperature and precipitation, GIMMS 3g, residual analysis

## Abstract

Vegetation in eastern Inner Mongolia grasslands plays an important role in preventing desertification, but mineral exploration has negative effects on the vegetation of these regions. In this study, the changing trend types of vegetation in eastern Inner Mongolia were analyzed using the normalized difference vegetation index (NDVI) time series from the Global Inventory Modeling and Mapping Studies (GIMMS) NDVI 3g dataset from 1982 to 2015. Meanwhile, changing trend and influencing factors of 25 large-scale mining areas before and after mining were explored with the methods of trend line, residual calculation, and correlation analysis. The vegetation coverage towards increasing in eastern Inner Mongolia decreased in the order of Tongliao > Hinggan League > Chifeng > Hulunbuir > Xilingol over the past 34 years. Vegetation showed a decreasing tendency in 40% mining areas, but an increasing tendency in 60% mining areas after mining. Vegetation change in Shengli No. 1 had a significant correlation with precipitation and human activities after mining. Except Shengli No. 1, an obvious correlation was found between vegetation change and precipitation in 45.83% mining areas after mining. Human activities had significant positive effects on vegetation growth in 25% mining areas. Significant negative effects of human activities were found in 8.34% mining areas, causing the vegetation degradation. However, there were 20.83% mining areas with vegetation changes not affected by precipitation and human activities.

## 1. Introduction

Grasslands in the world are mainly located in Eurasian Steppe, North American Steppe, and South American Steppe [1]. Meanwhile, there are a lot of coal resources in these regions. For instance, coal reserves are estimated at 122.4 million tons in the Powder River Basin, Wyoming [2]. In China, the proved coal reserves are more than 800 million tons, forming two large coal-electricity bases in eastern Inner Mongolia [3]. However, the exploitation and utilization of mineral resources have changed the material cycle and energy flow of mining area ecosystem, resulting in serious vegetation degradation and environmental pollution [4]. Therefore, there has been increasing attention given to explore vegetation change in grassland mining areas [5,6].

Grassland vegetation plays a pivotal role in the Earth’s material and energy exchange, which is the most sensitive part of ecosystems to climate change in arid and semi-arid environments [7]. Climate change is an important driving factor for terrestrial vegetation variation, providing required heat and water for vegetation growth. Suitable temperature can promote stems and leaves growth by vegetation transpiration [8]. In addition, abundant precipitation of growing season has a positive impact on vegetation root development [9]. However, there is a negative effect on vegetation growth from extreme climate (e.g., drought, frost risk, and heat stress) [10]. Renne et al. [11] reported that high temperature and extreme precipitation lead to an increase in tree and big sagebrush mortality. Hence, the influences of climate factors on vegetation growth cannot be ignored. 

Mining is well-known as one of the most aggressive human disturbances leading to massive and irreversible damages to natural ecosystems [12]. At present, many observers believe extensive vegetation degradation is generally a likely consequence of coal mining activities [13,14]. Mining significantly affects topography and surface cover, air quality, water quality, vegetation development, and soil physical and chemical properties [15]. Existing research has shown that there is an obvious correlation among vegetation change and soil properties (e.g., soil texture, moisture, and pH) [16,17]. Ecological restoration measures taken by humans have prompted the vegetation restoration in mining areas [18].

Grassland in eastern Inner Mongolia is located in the northern sand control area of the national ecological security strategy of “Two Screens and Three Belts”, with a fragile ecological environment. It has experienced a history of coal mining that dates back more than 100 years. The National Energy Administration announced that there were 56 coal mines with an annual production capacity over 300,000 tons on 31 Dec. 2018 in eastern Inner Mongolia (Hulunbuir, Xilingol, Chifeng, Tongliao, and Hinggan League) [19]. With the continuous expansion of mining area, the pressure of regional ecological environment tends to increase. More attention needs to be paid to the contradiction between coal mining and vegetation. 

Many scholars have noticed vegetation change in Inner Mongolia [20,21]. Advanced Very High Resolution Radiometer (AVHRR) NDVI data, especially the Global Inventory Modeling and Mapping Studies (GIMMS) NDVI 3g data, has been widely used to analyze the vegetation change in Inner Mongolia with its advantage of long-time series and wide coverage [22,23]. Vegetation change of eastern Inner Mongolia from 1981 to 2015 can be obtained from the previous study. However, vegetation change in mining areas in eastern Inner Mongolia is unclear based on the present study. Is the vegetation change trend consistent before and after mining? Does mining affect vegetation coverage? How do climate factors influence vegetation coverage? The objectives of this study were: (1) to investigate an overview of vegetation change in eastern Inner Mongolia; (2) to analyze changing trend of vegetation before and after mining in large-scale mining area; (3) to explain the relationship between the mining area and its buffer zones; and (4) to obtain influencing factors of vegetation change after mining in these mining areas.

## 2. Materials and Methods

### 2.1. Study Area

**Figure 1:**
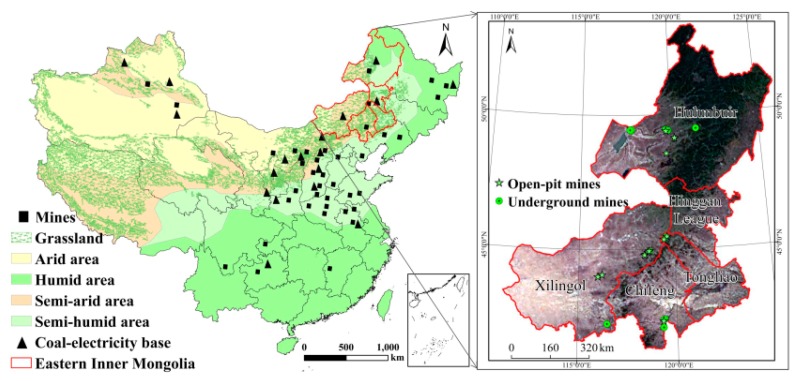
Location of large-scale mines in eastern Inner Mongolia.

The regions of eastern Inner Mongolia include Hulunbuir, Xilingol, Chifeng, Tongliao, and Hinggan League (latitude 111.2°–126°N, longitude 41.3°–53.3°E) (Figure 1) [24]. There is a semi-arid and arid climate, and precipitation is in the range of 200–400 mm p.a. The tempo-spatial distribution of precipitation is extremely uneven, mostly concentrated in summer, and it is easy to form floods and soil erosion. The area of grassland and forest are 1.98 × 10^7^ and 3.43 × 10^7^ hm^2^, covering approximately 30.31% and 50.66% of eastern Inner Mongolia, respectively [25]. The notice on adjusting the scale standards for production and construction of some mines (2004, No. 208) from the Ministry of Natural Resources of the People’s Republic of China stipulated the scale of production and construction of large-scale mines. The annual production capacity of a large-scale mine is over 4,000,000 tons for open-pit mine and 1,200,000 tons for underground coal mine. There are 25 large-scale mines in eastern Inner Mongolia, as shown in Table 1.

### 2.2. Data

#### 2.2.1. Remote Sensing Vegetation Data

This study used the Global Inventory Modeling and Mapping Studies (GIMMS) NDVI 3g. v1 (third generation version 1) bimonthly products with spatial resolution of 8 km × 8 km [51]. They were downloaded from https://ecocast.arc.nasa.gov/data/pub/gimms/3g.v1/00FILE-LIST.txt and covered a period from July 1981 to December 2015. These NDVI datasets have been corrected for calibration, viewing geometry, volcanic aerosols, and other effects that are not related to vegetation variation. These data contain global geographical projections (Geographic, WGS 1984). The GIMMS products are 16-day maximum value composite (MVC) bimonthly global NDVI product generated from AVHRR data. The datasets are in NetCDF format. We translated these datasets into GeoTIFF format using MatlabR2014a. NDVI datasets from 1982 to 2015 were selected in this study.

#### 2.2.2. Vector Boundary Data

The city boundaries were from the 1:100 million vector map data of the National Catalogue Service *for* Geographic Information downloaded from http://www.webmap.cn/commres.do?method=result100W. The mining area boundary was obtained by its inflection points, geographic coordinates, and Google Earth images, which showed in the references of [25,26,27,28,29,30,31,32,33,34,35,36,37,38,39,40,41,42,43,44,45,46,47,48,49]. The buffer boundary (e.g., 10, 20, 30, 40, and 50 km) was completed based on their mining area boundary by buffer analysis of ArcGIS software. GIMMS NDVI 3g data were clipped using city and mining area boundaries.

#### 2.2.3. Climate Observation Data

Daily average temperature (°C) and rainfall (mm) data from July 1982 to December 2015 were provided by the National Oceanic and Atmospheric Administration (NOAA) downloaded from https://gis.ncdc.noaa.gov/maps/ncei/cdo/daily. There are 16 monitoring sites in eastern Inner Mongolia, consisting of three in Hulunbuir, seven in Xilingol, three in Chifeng, two in Tongliao, and one in Hinggan League. The annual average temperature and precipitation of growing season at each monitoring site from 1982 to 2015 were calculated by its daily average temperature and rainfall from April to October each year. The space distribution of temperature and precipitation each year in eastern Inner Mongolia was realized by Kriging interpolation of ArcGIS software.

### 2.3. Methods

#### 2.3.1. Maximum Value Composites

Considering the demand of vegetation growth for temperature and precipitation, we selected April to October per year as the vegetation growing season. The Maximum Value Composite (MVC) method was used to obtain the max monthly NDVI (MNDVI). In addition, the max NDVI of growing season each year (GNDVI) was calculated based on the *MNDVI* from April to October [22]. In this study, GNDVI was used to reflect the vegetation coverage. *MNDVI* and *GNDVI* were computed using the following equations:

(1) $$MNDVI_{ij}=MAX(NDVI_{ij}{}_{1},NDVI_{ij}{}_{2})$$

(2) $$GNDVI_{i}=MAX(MNDVI_{i}{}_{4},MNDVI_{i}{}_{5},\cdots ,MNDVI_{i}{}_{9},MNDVI_{i}{}_{10})$$

where i is the series number of year (i=1,2,⋯,34), j is the series number of month (j=1,2,⋯,12), $MNDVI_{\mathrm{ij}}$ is the max *NDVI* for the *j*th month of the *i*th year, $NDVI_{\mathrm{ij}}{}_{1}$ is max *NDVI* for the first half of the *j*th month of the *i*th year, $NDVI_{\mathrm{ij}}{}_{2}$ is max *NDVI* for the second half of the *j*th month of the *i*th year, and $GNDVI_{i}$ is the max *NDVI* for the *i*th year.

#### 2.3.2. Trend Line Analysis

We adopted the ordinary least-squares (OLS) approach to determine the changing trend of GNDVI, precipitation, temperature, and residual from 1982 to 2015 [22]. The slope was calculated using the following equations:

(3) $$slope=\frac{n\cdot \sum_{i=1}^{n}\left(i\cdot x_{i}\right)-\sum_{i=1}^{n}i\sum_{i=1}^{n}x_{i}}{n\cdot \sum_{i=1}^{n}i^{2}-{\left(\sum_{i=1}^{n}i\right)}^{2}}$$

where *n* represents the total number of years (n=34), i is the series number of year (i=1,2,⋯,34), and *x_i_* is the value (GNDVI, precipitation, temperature, and residual) of year *i*. A slope greater than 0 indicates an increasing trend, and one less than 0 shows a decreasing trend.

The significance degree of changing trend is determined by the correlation coefficient *r*. *r* was calculated using the following equations:

(4) $$r=\sqrt{\frac{n\cdot \sum_{i=1}^{n}i^{2}-{\left(\sum_{i=1}^{n}i\right)}^{2}}{n\cdot \sum_{i=1}^{n}x_{i}{}^{2}-{\left(\sum_{i=1}^{n}x_{i}\right)}^{2}}}\cdot slope$$

*r* greater than 0 indicates an increasing trend, and r less than 0 shows a decreasing trend.

In addition, the significance level of change trend was assessed using the t-test, and the *p*-value was adopted to reflect the significance level. The changing types and significance levels are shown in Table 2 [52]. t was calculated using the following equations:

(5) $$t=\frac{r}{\sqrt{1-r^{2}}}\sqrt{n-2}$$

#### 2.3.3. Correlation Analysis

Correlation coefficients were calculated between mining area and its buffer zones for vegetation variation. In addition, we applied correlation analysis to explore influencing factors of vegetation variation. Pearson correlation coefficients were calculated among the GNDVI and precipitation and temperature from 1982 to 2015.

#### 2.3.4. Residual Analysis

We adopted pixel-based residual trend analysis to distinguish the human-induced GNDVI changes from the changes induced by climate factors. We defined the residual as the difference between the observed GNDVI and the predicted GNDVI [21]. The equation is shown as follows:

(6) $$GNDVI_{P}=a\times T+b\times P+c$$

(7) $$GNDVI_{r}=GNDVI_{O}-GNDVI_{P}$$

where $GNDVI_{P}$ is the observed *GNDVI*, *a* and *b* are the regression coefficients, *c* is constant, and *T* and *P* are the temperature and precipitation, respectively. $GNDVI_{r}$ is the *GNDVI* residuals. $GNDVI_{O}$ is the predicted *GNDVI*.

#### 2.3.5. Statistical Analysis and Spatial Distribution Maps

GNDVI in mining areas and buffer zones and GNDVI variances were achieved by using Excel software (Version 14, Microsoft, Inc., Redmond, Washington, DC, USA). In this study, the weight of GNDVI in mining area is the ratio of mining area to the area of all pixels covered. In addition, GNDVI in buffer zones are based on their corresponding ratios. Ordinary kriging (OK) interpolation was performed on the climate factors and the trend line analysis using ArcGIS software (Version 10.2, ESRI, Inc., Redlands, CA, USA). Then, correlation analysis was achieved by using IBM SPSS statistics software (Version 19.0, Armonk, NY, USA).

## 3. Results

### 3.1. Tempo-Spatial Change of Vegetation Coverage in eastern Inner Mongolia

**Figure 2:**
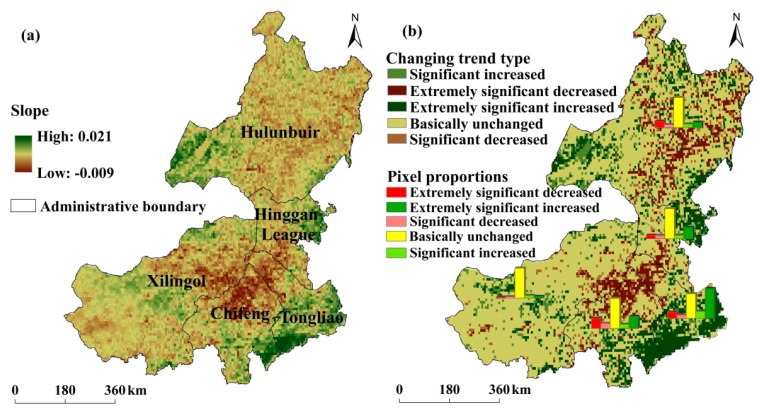
GNDVI slope and changing trend of vegetation coverage in eastern Inner Mongolia from 1982 to 2015: (**a**) GNDVI slope; and (**b**) Changing trend type.

The GNDVI slope in eastern Inner Mongolia is shown in Figure 2. Overall, GNDVI slope values were in the range of −0.009 to 0.021, exhibiting an obvious tempo-spatial difference from 1982 to 2015 (Figure 2a). Changing trend types and their proportions are shown in Figure 2b and Table 3, respectively. Vegetation coverage of 9.97% pixels mainly located in central Hulunbuir, northern Chifeng, and northwest Tongliao presented an extremely significant decreasing trend. An extremely significant increasing trend was found in vegetation coverage of 14.22% pixels distributed in western Hulunbuir, southeast Chifeng and Tongliao, and eastern Hinggan League. There were 62.59% pixels in eastern Inner Mongolia and their vegetation coverage remained unchanged over the past 34 years.

In Hulunbuir, GNDVI slope values ranged from −0.008 to 0.021. Vegetation coverage of 61.56% pixels has no significant change. A decreasing trend was found in vegetation coverage of 19.71% pixels in Ewenki Autonomous Banner, Yakeshi, and Oroqen Autonomous Banner. Vegetation coverage of 18.73 pixels showed an increasing trend, and they were located in Xin Barag Left Banner, Xin Barag Right Banner, Argun, and ZhaLanTun. In Xilingol, GNDVI slope values were in the range of −0.005 to 0.005. An insignificant change was seen in vegetation coverage of 79.96% pixels. There was a decreasing trend of vegetation coverage of 7.31% pixels distributed in Sonid Right Banner and West Ujimqin Banner. Vegetation coverage of 12.73% pixels tended to increase and they were located in Xilin Hot, Abag Banner, and Sonid Left Banner. In Chifeng, GNDVI slope values ranged from −0.006 to 0.007. The proportion of pixels with no change was 48.7%, suggesting that vegetation coverage of these pixels was unchanged over the past 34 years. Vegetation coverage of 26.43% pixels increased and they were distributed in Aohan Banner, Wengniute Banner, Ningcheng, and Kalaqin Banner. In Tongliao, GNDVI slope values were in the range of −0.006 to 0.008. Vegetation coverage of 51.85% pixels in Naiman Banner, Kulun Banner, Kailu, Horqin Left Wing Rear Banner, and Horqin Left Wing Middle Banner exhibited an increasing trend. In Hinggan League, GNDVI slope values ranged from 0.004 to 0.007. There were 57.55% pixels with no change. The proportion of increased pixels (including significant increased and extremely significant increased) decreased in the order of Tongliao > Hinggan League > Chifeng > Hulunbuir > Xilingol, suggesting that the change of vegetation in Tongliao is better than in other cities [22].

### 3.2. Vegetation Coverage Change of 25 Large-Scale Mining Areas in eastern Inner Mongolia

**Figure 3:**
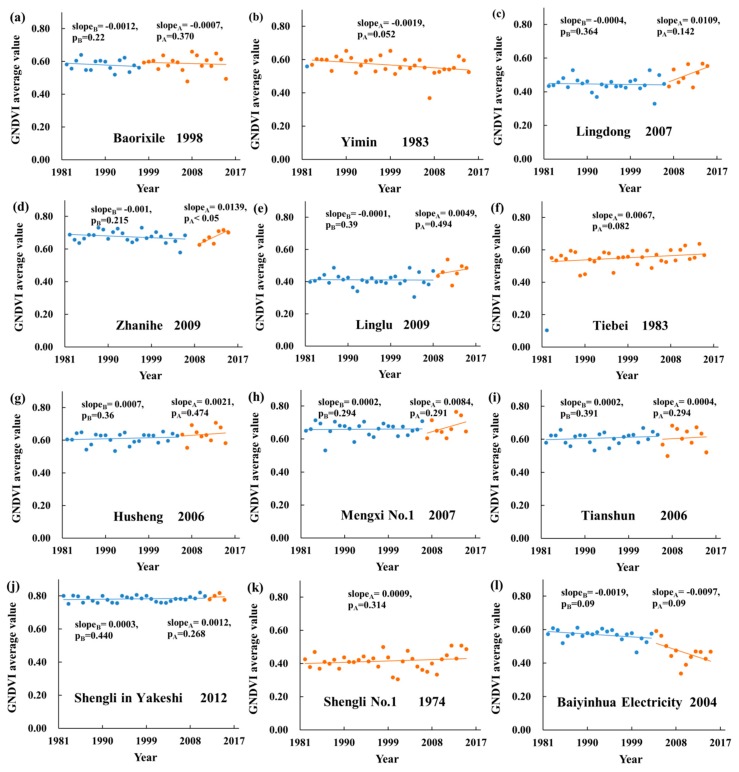

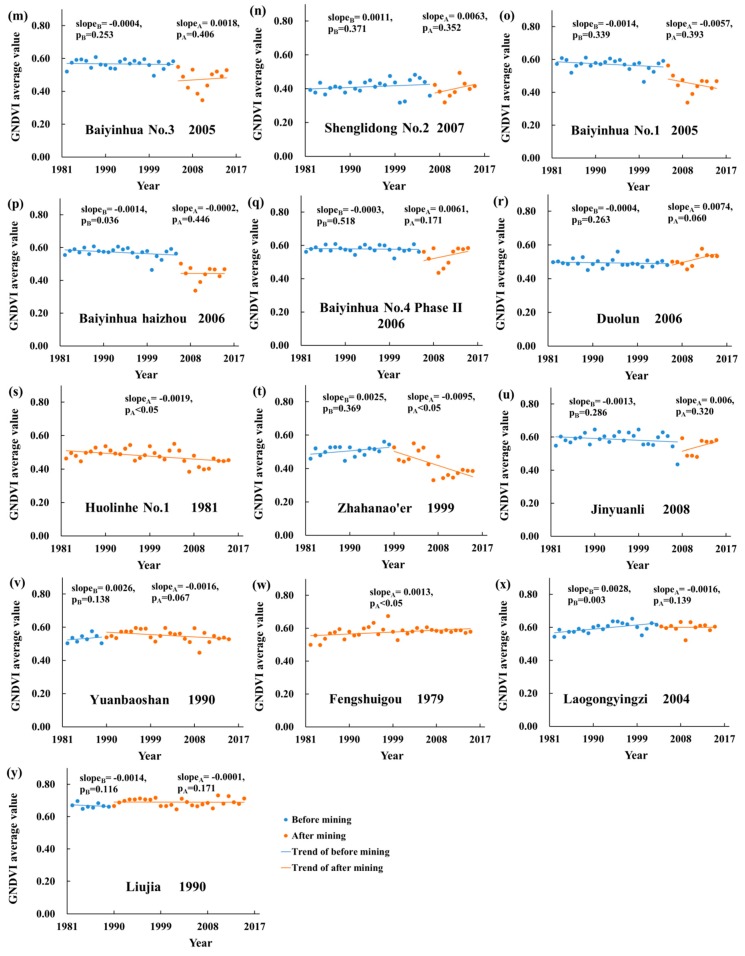
GNDVI of 25 large-scale mining areas: (**a**) Baorixile; (**b**) Yimin; (**c**) Lingdong; (**d**) Zhanihe; (**e**) Linglu; (**f**) Tiebei; (**g**) Husheng; (**h**) Mengxi No. 1; (**i**) Tianshun; (**j**) Shengli; (**k**) Shengli No. 1; (**l**) Baiyinhua Electricity; (**m**) Baiyinhua No. 3; (**n**) Shenglidong No. 2; (**o**) Baiyinhua No. 1; (**p**) Baiyinhua Haizhou; (**q**) Baiyinhua No. 4 PhaseⅡ; (**r**) Duolun; (**s**) Huolinhe No. 1; (**t**) Zhahanao’er; (**u**) Jinyuanli; (**v**) Yuanbaoshan; (**w**) Fengshuigou; (**x**) Laogongyingzi; and (**y**) Liujia. slope_B_, and p_B_ means before mining; slope_A_, and p_A_ means after mining.

Figure 3 shows the GNDVI before and after mining in 25 large-scale mining areas from 1982 to 2015. In Hulunbuir, for Baorixile, vegetation coverage showed insignificant slope values of decrease at −0.0012 per year (*p* = 0.22) and −0.0007 per year (*p* = 0.370) before and after mining (Figure 3a). For Yimin, there was an insignificant decrease trend of vegetation coverage with the slope value of −0.0019 per year (*p* = 0.052) after mining (Figure 3b). In terms of Lingdong and Linglu, an insignificant decrease trend was found in vegetation coverage with their slope values of −0.0004 per year (*p* = 0.364) and −0.001 per year (*p* = 0.39) before mining, but an insignificant increase trend was seen in vegetation coverage with their slope values of 0.0109 per year (*p* = 0.142) and 0.0049 per year (*p* = 0.494) after mining (Figure 3c,e). For Zhanihe, vegetation coverage had a significant increasing tendency after mining (Figure 3d), with the slope value of 0.0139 per year (*p* < 0.05). For Tiebei, vegetation coverage exhibited an insignificant slope value of increase at 0.0067 per year (*p* = 0.082) (Figure 3f). In terms of Husheng, Mengxi No. 1, Tianshun, and Shengli in Yakeshi, there was an insignificant increasing trend of vegetation coverage with their slope values above 0 per year before and after mining (Figure 3g–j). In Xilingol, for shengli No. 1, vegetation coverage presented an insignificant increasing tendency (Figure 3k), with the slope value of 0.0009 per year (*p* = 0.314) after mining. In terms of Baiyinhua Electricity, Baiyinhua No. 1, and Baiyinhua Haizhou, an insignificant decreasing tendency was found in vegetation coverage with their slope values below 0 per year before and after mining (Figure 3l,o,p). For Shenglidong No. 2, vegetation coverage showed an insignificant slope values of increase at 0.0011 per year (*p* = 0.371) and 0.0063 per year (*p* = 0.352) before and after mining (Figure 3n). For Baiyinhua No. 3, Baiyinhua No. 4 Phase Ⅱ, and Duolun, vegetation coverage presented an insignificant slope values of decrease below 0 per year before mining, but increase above 0 per year after mining (Figure 3m,q,r). In Tongliao, for Huolinhe No. 1 and Zhahanao’er, a significant decreasing tendency was found in vegetation coverage with their *p*-values below 0.05 after mining (Figure 3s,t: slope = −0.0019 and −0.0095). For Jinyuanli, there was an opposite changing trend in vegetation coverage before and after mining, and the changing trends were insignificant (Figure 3u). In Chifeng, for Yuanbaoshan, Laogongyingzi, and Liujia, vegetation coverage showed an insignificant slope values of decrease after mining, with their slope values below 0 (Figure 3v,x,y: *p* = 0.067, 0.139 and 0.171). For Fengshuigou, a significant increasing tendency was seen in vegetation coverage after mining (Figure 3w: slope = 0.0013, *p* < 0.05).

### 3.3. Vegetation Coverage Correlation between 25 Large-Scale Mining Areas and Its Buffer Zones

**Figure 4:**
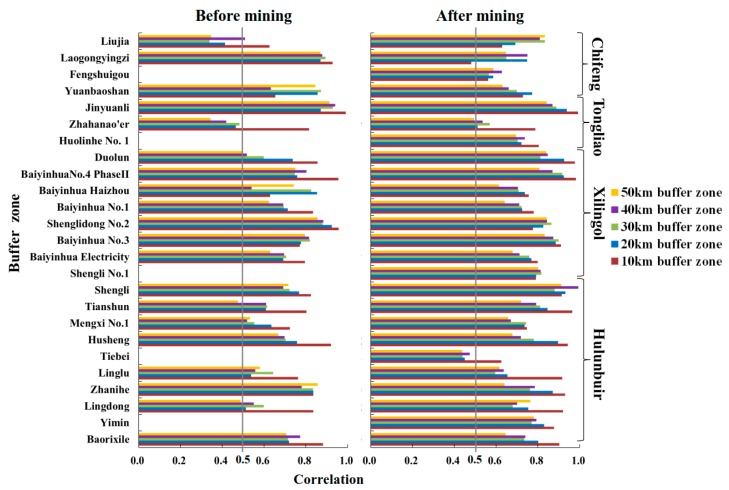
Correlation of GNDVI between mining area and its buffer zones before and after mining.

Correlation analysis of GNDVI between 25 large-scale mining areas and its buffer zones is exhibited in Figure 4. In general, before and after mining, there was a positive correlation of vegetation coverage between mining area and its buffer zone for each mining area. For 16 mining areas, greater correlation coefficients were found in vegetation coverage between mining area and its 10 km buffer zones. Before mining, for Lingdong, Tianshun and Duolun, the correlation coefficients of vegetation coverage between mining areas and their 50 km buffer zones were less than 0.5 (*p* < 0.05). For Zhahanao’er, except 10 buffer zone, the correlation coefficients of vegetation coverage were smaller than 0.5 (*p* < 0.05) between mining area and other buffer zones. For Liujia, smaller correlation coefficients were found in vegetation coverage among mining area and its 20, 30, and 50 km buffer zones. However, for the above five mining areas, a significant growth was seen in the correlation coefficients in vegetation coverage between mining areas and their buffer zones after mining. For Tiebei, except 10 km, there were poor correlation coefficients between mining area and its buffer zones after mining.

### 3.4. Correlation between Vegetation Coverage and Climatic Factors of 25 Large-Scale Mining Areas

The correlation coefficients among GNDVI and temperature and precipitation of each mining area before and after mining are shown in Table 4. In general, except Yimin and Huolinhe No. 1, an insignificant relationship was found between GNDVI and temperature before and after mining. This suggests that temperature has little effect on vegetation change. For Mengxi No. 1 and Shenglidong No. 2, precipitation made a greater contribution to vegetation change from 1982 to 2015 (*p* < 0.05 and *p* < 0.01). In Shengli, for Yakeshi, Shengli No. 1, Baiyinhua No. 3, Baiyinhua No. 1, Baiyinhua Haizhou, Baiyinhua No. 4 Phase Ⅱ, Duolun, and Yuanbaoshan, precipitation played an important role in vegetation change after mining (*p* < 0.05 and *p* < 0.01). For, Baorixile, Linglu, and Tianshun, there was a significant relationship between GNDVI and precipitation before mining, but an insignificant relationship was seen after mining. In eight mining areas, temperature and precipitation had significant impacts on vegetation change.

### 3.5. Correlation between Vegetation Coverage and Human Factors of 25 Large-scale Mining Areas

**Figure 5:**
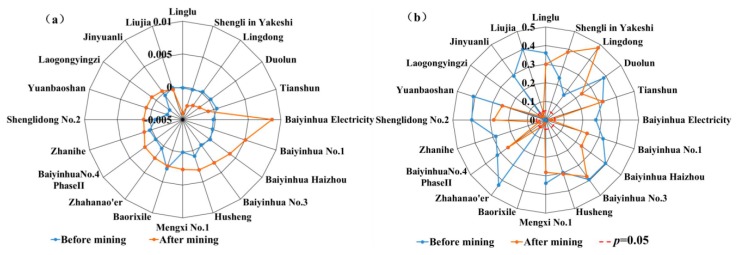
Residual slope from 1982 to 2015: (**a**) slope; and (**b**) *p*-value.

Residual reflects the impact produced by human activities. Figure 5 shows the residual slope values and t-test. For Baorixile and Laogongyingzi, human activities showed a significant influence on the vegetation change in the past 34 years (*p* < 0.05), especially promoting vegetation growth after mining. For Baiyinhua Electricity, Zhahanao’er, Zhanihe, and Jinyuanli, there was a significant increasing residual trend (slope >0, *p* < 0.05) after mining, indicating human activities have a positive effect on vegetation growth. For Liujia, we found a decreasing residual trend (slope <0, *p* < 0.05) after mining, which suggests that the degradation of vegetation was caused by human activities. In addition, in terms of Shengli No 1 and Fengshuigou, human activities played an important role in the degradation of vegetation with the slope values of *−*0.0003 (*p* < 0.05) and *−*0.001 (*p* < 0.05) after mining. However, for the remaining 15 mining areas, human activities presented an insignificant influence on the vegetation change from 1982 to 2015 (*p* > 0.05).

## 4. Discussion

### 4.1. Vegetation Change Before and After Mining in Mining Mrea in Eastern Inner Mongolia

**Figure 6:**
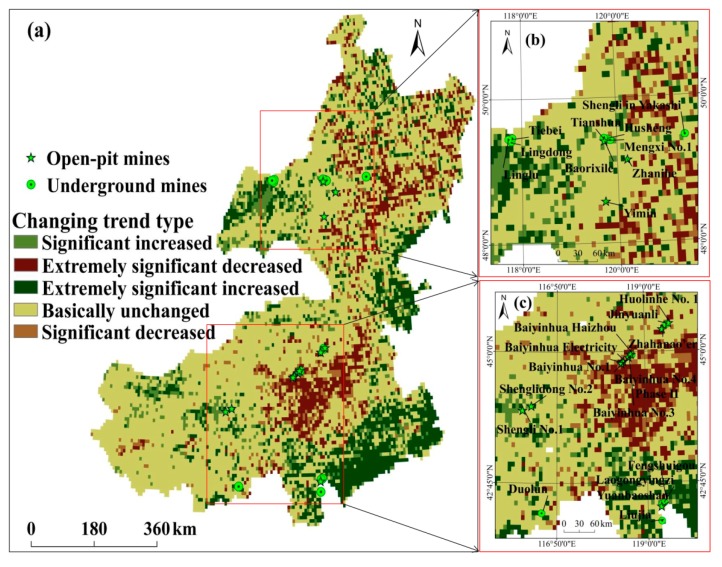
Changing trend of 25 large-scale mining areas from 1982 to 2015.

**Figure 7:**
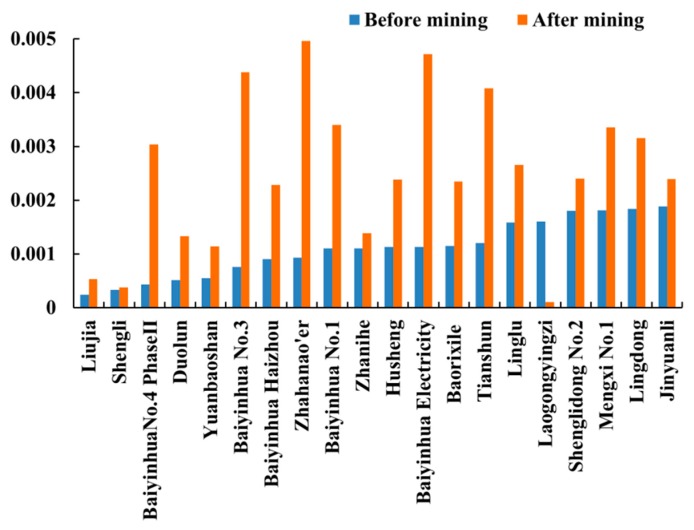
Variance of GNDVI in mining areas before and after mining.

Over the past 34 years, vegetation change in more than half of eastern Inner Mongolia was not obvious. Similar findings were reported by Chen et al. and Mu et al. [53,54]. However, significant changes were found in vegetation coverage in mining areas. In general, from 1982 to 2015, vegetation change in most mining areas in Hulunbuir and Chifeng tended to increase, but in Xilingol and Tongliao tended to decrease (Figure 6). In terms of vegetation change, seven mining areas presented a decreasing tendency before mining, but an increasing tendency after mining. Meanwhile, a more significant growth was found after mining in seven mining areas. Moreover, there were three mining areas with a less obvious decreasing tendency after mining. This suggests that vegetation change in most mining areas in eastern Inner Mongolia showed an increasing trend after mining. Changing degree of vegetation coverage can be expressed by the variance of GNDVI. As shown in Figure 7, except Laogongyingzi, vegetation coverage exhibited more obvious fluctuations after mining. This also reflected the effects of human activities on the vegetation growth in mining areas.

### 4.2. Response of Vegetation Change to Climatic Factors in Mining Area

Existing research has shown that precipitation during the growing season plays a more important role than temperature in vegetation growth in semi-arid and arid regions [55]. As shown in Table 4, vegetation changes were affected significantly by temperature during the growing season in only two mining areas. For Yimin, vegetation remained basically unchanged from 1982 to 2015 (Figure 6). The vegetation fluctuation after mining was the result of change in temperature and precipitation, with little influence from mining activities [56]. For Huolinhe No. 1, Li et al. [57] and Wu et al. [58] reported that temperature change had a significant impact on agricultural production in the past 50 years. There was a significant positive correlation between vegetation change and precipitation in 14 mining areas. These mining areas were located in Hulunbuir and Xilingol. Meng et al. [21] found that there was a significant change in precipitation during the growing season (spring, summer, and autumn) in Hulunbuir and Xilingol. In addition, Zhang et al. [59] and Hang et al. [60] reported similar findings. In addition, the weakening correlation between vegetation and temperature may be due to drought in the Northern Hemisphere [60].

### 4.3. Do Human Activities Affect Vegetation Growth in Mining Area?

Figure 5a shows that human activities had a positive or negative impact on vegetation growth. Vegetation degradation in a quarter of mining areas was caused by human activities after mining. Zhang et al. [13] and Bao et al. [22] reported that underground and open-pit mining results in vegetation degradation in mining areas. However, in this study, human activities promoted vegetation growth in approximately three quarters of mining areas after mining. This could be explained by the application of concurrent mining and reclamation. Remediation technologies mainly included slope treatment, soil fertilization, and bioremediation. For instance, for Baorixile, from 1998 to 2000, the GNDVI increasing tendency indicated that human activities had little effect on vegetation coverage in the early stage of mining [61]. The GNDVI in 2007 was lowest, which might have been caused by the reconstruction and expansion project in 2006 [62]. Meanwhile, a near-term governance planning (2010–2013) was formulated and implemented to protect the environment by applying slope treatment and soil maturation [63]. For Zhanihe, strip controlled mining technology was used for slope stabilizing in 2013, which prevented the vegetation degradation from 2013 to 2014 [64]. For Zhahanao’er, slope protection technology by spraying vegetation on carrying soil was applied in 2009, leading to an increase in vegetation coverage between 2010 and 2011 [65]. In addition, selecting proper plants was crucial to vegetation restoration in mining area. *Leymus chinensis* and *hippophae rhamnoides* were always selected and planted in Baorixile with developed root and drought tolerance [63]. Management of replanting vegetation played an active role in the ecological remediation of mining area [66]. Hence, we should take similar and suitable measures to protect vegetation for Linglu, Lingdong, Duolun, Tianshun, and Shengli in Yakeshi.

## 5. Conclusions

From 1982 to 2015, vegetation change in more than half of eastern Inner Mongolia was insignificant, but significant changes were found in mining areas. Vegetation coverage of 60% of large-scale mining areas showed an increasing tendency after mining. During the growing season, the responses of vegetation change to precipitation were obvious in mining areas. Human activities in mining areas promoted vegetation growth. These findings help to better understand the effects of active human activities on vegetation growth in mining areas, and to pay more attention to vegetation degraded areas. We will explore other influencing factors (e.g., land use change, overgrazing, and soil condition) in the future.

## Figures and Tables

**Table 1:** Large-scale mines in eastern Inner Mongolia.

Mines	Recovery Method	Location	Annual Production Capacity/t	Construction Time/Year
Baorixile [26]	Open-pit	Chenbarhu Banner, Hulunbuir	3500	1998
Yimin [27]	Open-pit	Ewenki Autonomous Banner, Hulunbuir	2200	1983
Lingdong [28]	Underground mining	Dalai Nur District, Hulunbuir	650	2007
Zhanihe [29]	Open-pit	Ewenki Autonomous Banner, Hulunbuir	600	2009
Linglu [30]	Underground mining	Dalai Nur District, Hulunbuir	390	2009
Tiebei [31]	Underground mining	Dalai Nur District, Hulunbuir	360	1983
Husheng [32]	Underground mining	Chenbarhu Banner, Hulunbuir	180	2006
Mengxi No. 1 [33]	Underground mining	Chenbarhu Banner, Hulunbuir	180	2007
Tianshun [34]	Underground mining	Chenbarhu Banner, Hulunbuir	120	2006
Shengli [35]	Underground mining	Yakeshi, Hulunbuir	120	2012
Shengli No. 1 [36]	Open-pit	Xilin Hot, Xilingol	2000	1974
Baiyinhua Electricity [37]	Open-pit	West Ujimqin Banner, Xilingol	1500	2004
Baiyinhua No. 3 [38]	Open-pit	West Ujimqin Banner, Xilingol	1400	2005
Shenglidong No. 2 [39]	Open-pit	Xilin Hot, Xilingol	1000	2007
Baiyinhua No. 1 [40]	Open-pit	West Ujimqin Banner, Xilingol	700	2005
Baiyinhua Haizhou [41]	Open-pit	West Ujimqin Banner, Xilingol	500	2006
Baiyinhua No. 4 PhaseⅡ [42]	Underground mining	West Ujimqin Banner, Xilingol	500	2006
Duolun [43]	Underground mining	Xilin Hot, Xilingol	120	2006
Huolinhe No. 1 [44]	Open-pit	Holingola, Tongliao	1800	1981
Zhahanao’er [45]	Open-pit	Jarud Banner, Tongliao	1800	1999
Jinyuanli [46]	Underground mining	Holingola, Tongliao	120	2008
Yuanbaoshan [47]	Open-pit	Yuanbaoshan District, Chifeng	800	1990
Fengshuigou [48]	Underground mining	Yuanbaoshan District, Chifeng	210	1979
Laogongyingzi [49]	Underground mining	Yuanbaoshan District, Chifeng	180	2004
Liujia [50]	Underground mining	Yuanbaoshan District, Chifeng	180	1990

**Table 2:** Classification of significant test results.

Changing Types	*Slope*	Significance Level *p*
Extremely significant decrease	slope<0	p<0.01
Significant decrease		0.01<p<0.05
Basically unchanged		p>0.05
Significant increase	slope>0	0.01<p<0.05
Extremely significant increase		p<0.01

**Table 3:** Changing trend types of vegetation coverage in eastern Inner Mongolia.

Changing Trend Types	Number of Pixels	Proportion
Extremely significant decreased	1095	9.97%
Significant decreased	627	5.71%
Basically unchanged	6873	62.59%
Significant increased	824	7.53%
Extremely significant increased	1562	14.22%

**Table 4:** Correlation coefficients between climate factors and GNDVI of 25 large-scale mining areas.

Mines	Before Mining		After Mining	
	Temperature	Precipitation	Temperature	Precipitation
Baorixile	0.291	0.563 *	−0.361	0.226
Yimin	−	−	−0.419 **	0.381 *
Lingdong	−0.001	0.147	−0.166	0.213
Zhanihe	−0.101	0.298	−0.264	0.729
Linglu	−0.058	0.51 **	−0.391	0.316
Tiebei	−	−	−0.195	0.108
Husheng	0.309	0.171	−0.582	0.547
Mengxi No. 1	0.176	0.512 **	−0.622	0.809 **
Tianshun	0.264	0.486 *	−0.448	0.383
Shengli in Yakeshi	−0.192	0.256	−0.198	0.389 *
Shengli No. 1	−	−	−0.240	0.665 **
Baiyinhua Electricity	−0.321	0.139	−0.427	0.552
Baiyinhua No. 3	−0.184	0.352	−0.562	0.685 *
Shenglidong No. 2	−0.039	0.431 *	−0.658	0.807 **
Baiyinhua No. 1	−0.288	−0.018	−0.433	0.691 *
Baiyinhua Haizhou	−0.096	0.282	−0.573	0.756 *
Baiyinhua No. 4 PhaseⅡ	−0.161	0.331	−0.536	0.641 *
Duolun	−0.196	0.200	−0.574	0.677 *
Huolinhe No. 1	−	−	−0.534 **	0.297
Zhahanao’er	0.108	−0.206	−0.307	0.474
Jinyuanli	−0.245	0.243	−0.177	0.587
Yuanbaoshan	−0.303	0.211	−0.365	0.543 **
Fengshuigou	−	−	−0.759	0.590
Laogongyingzi	−0.087	0.387	−0.344	0.105
Liujia	−0.093	0.015	−0.194	0.158

Notes: * and ** represent *p* < 0.05 and *p* < 0.01, respectively. – represents that there is no value.

## References

[1] Hou X.Y. (2014). Thinking and Practice Based on the Grassland in Northern China and the Protection and Development of the Eurasian Steppe. Chin. J. Grassl..

[2] Hefferna E.L., Coatesb D.A. (2004). Geologic History of Natural Coal-bed Fires, Powder River basin, USA. Int. J. Coal Geol..

[3] Yuan J.X., Tian X.C. (2015). Investigation on Production and Management of Coal Enterprises under the Background of Overcapacity—A case of Inner Mongolia. North. Econ..

[4] Fu X., Ma M.F., Jiang P., Quan Y. (2017). Spatiotemporal vegetation dynamics and their influence factors at a large coal-fired power plant in Xilinhot, Inner Mongolia. Int. J. Sustain. Dev. World.

[5] Stearns M., Tindall J., Cronin G., Friedel M.J., Bergquist E. (2005). Effects of Coal-bed Methane Discharge Waters on the Vegetation and Soil Ecosystem in Powder River Basin, Wyoming. Water Air Soil Poll..

[6] Sha Z.Y., Wang Y.W. (2019). Detecting grassland cover changes through spatiotemporal outlier analysis using remotely sensed time-series data: A case study from Xilingol, China. Geocarto Int..

[7] Wang B., Xu G.C., Li P., Li Z.B., Zhang Y.X., Cheng Y.T., Jia L., Zhang J.X. (2020). Vegetation dynamics and their relationships with climatic factors in the Qinling Mountains of China. Ecol. Indic..

[8] Du S.H., Fang S.Z. (1984). Relationship between plant transpiration, mercury uptake rate and temperature. Environ. Sci..

[9] Li G., Sun S.B., Han J.C., Yan J.W., Liu W.B., Wei Y., Lu N., Sun Y.Y. (2019). Impacts of Chinese Grain for Green program and climate change on vegetation in the Loess Plateau during 1982–2015. Sci. Total Environ..

[10] Li P., Sheng M.Y., Yang D.W., Tang L.H. (2019). Evaluating flood regulation ecosystem services under climate, vegetation and reservoir influences. Ecol. Indic..

[11] Renne R.R., Schlaepfer D.R., Palmquist K.A., Bradford J.B., Burke I.C., Lauenroth W.K. (2019). Soil and stand structure explain shrub mortality patterns following global change-type drought and extreme precipitation. Ecology.

[12] Hou X.Y., Liu S.L., Zhao S., Dong S.K., Sun Y.X., Beazley R. (2019). The alpine meadow around the mining areas on the Qinghai-Tibetan Plateau will degenerate as a result of the change of dominant species under the disturbance of open-pit mining. Environ. Pollut..

[13] Zhang D., Fan G., Ma L., Wang X. (2011). Aquifer protection during longwall mining of shallow coal seams: A case study in the Shendong Coalfield of China. Int. J. Coal Geol..

[14] Woodworth M.D. (2015). China’s coal production goes west: Assessing recent geographical restructuring and industrial transformation. Prof. Geogr..

[15] Hindersmann B., Achten C. (2018). Urban soils impacted by tailings from coal mining: PAH source identification by 59 PAHs, BPCA and alkylated PAHs. Environ. Pollut..

[16] Wen X., Deng X.Z., Zhang F. (2019). Scale effects of vegetation restoration on soil and water conservation in a semi-arid region in China: Resources conservation and sustainable for management. Resour. Conserv. Recy..

[17] Klichowska E., Nobis M., Piszczek P., Blaszkowski J., Zubek S. (2019). Soil properties rather than topography, climatic conditions, and vegetation type shape AMF-feathergrass relationship in semi-natural European grasslands. Appl. Soil Ecol..

[18] Li P.F., Zhang X.C., Hao M.D., Cui Y.X., Zhu S.L., Zhang Y.J. (2019). Effects of Vegetation Restoration on Soil Bacterial Communities, Enzyme Activities, and Nutrients of Reconstructed Soil in a Mining Area on the Loess Plateau, China. Sustainability.

[19] National Energy Administration National Coal Mine Production Capacity (Coal Mines in Production). http://zfxxgk.nea.gov.cn/auto85/201903/t20190326_3637.htm.

[20] Zhang R., Ouyang Z.T., Xie X., Guo H.Q., Tan D.Y., Xiao X.M., Qi J.G., Zhao B. (2016). Impact of Climate Change on Vegetation Growth in Arid Northwest of China from 1982 to 2011. Remote Sens..

[21] Meng M., Huang N., Wu M.Q., Pei J., Wang J., Niu Z. (2019). Vegetation Change in Response to Climate Factors and Human Activities on the Mongolian Plateau. PeerJ.

[22] Bao Y., Tian Y., Liu C.X., Fan W.Y., Fu X. (2018). Spatial and Temporal Variation Analysis of Vegetation Greenness in Grassland of Eastern China and Its Response on the Construction of Coal and Electricity Base. Acta Ecol. Sin..

[23] Tong S.Q., Zhang J.Q., Bao Y.H., Lai Q., Lian X., Li N., Bao Y.B. (2018). Analyzing vegetation dynamic trend on the Mongolian Plateau based on the Hurst exponent and influencing factors from 1982–2013. J. Geogr. Sci..

[24] Liu G. (2016). Study on Economic Development of Eastern Inner Mongolia under the Perspective of Regional Intergration. Ph.D. Thesis.

[25] Ministry of Natural Resources of the People’s Republic of China Land-use Type in Inner Mongolia Autonomous Region in 2016. http://tddc.mnr.gov.cn/to_Login.

[26] Inner Mongolia Autonomous Region Environmental Science Research Institute (2007). Environmental Impact Report of Open-Pit Coal Mine Reconstruction and Expansion Project of Shenhua Group Corporation Limited.

[27] Zhongyu Assets Evaluation Co., Ltd. (2009). Open-Pit Mining Right Evaluation Report of Huaneng Yimin Coal & Electricity Co., Ltd..

[28] Li Q. (2014). Research on Geological Environment Protection and Control of Lingdong Mine. Master’s Thesis.

[29] Yang H.H. (2010). The Stability Study on Composite Slope Consisted of the Stope and the South Dump in Zhanihe Open-Pit. Master’s Thesis.

[30] China Coal Research Institute (2019). Environmental Impact Report of Linglu Coal Mine Capacity Increase Project of China Coal Research Institute.

[31] Zhang Y.J. (2005). Predicting Study on Inrush of Sand and Overburden Failure of the Full Mechanized Top-Coal Caving under Loosening Sandstone Aquifer in the Tie-Bei Coal Mine. Master’s Thesis.

[32] Hulunbuir Environmental Supervision Company (2011). Environmental Supervision Report of 1.2 Million Tons Upgrade Project of Husheng Coal Mine of Hulunbuir Husheng Mining Co., Ltd..

[33] Liaoning Huanyu Mining Consulting Co., Ltd. (2014). Summary of the Mining Right Evaluation Report of Mengxi No.1 Mine of Hulunbuir Mengxi Coal Industry Co., Ltd..

[34] Inner Mongolia Autonomous Region Land and Resources Department Notice on the Public Selection and Evaluation Agency Undertaking Eight Mining Rights Assessment Projects Such as The Lijinshan Iron Mine in Ejina Banner, Inner Mongolia. http://zrzy.nmg.gov.cn/zwgk/tztg/201005/t20100504_30973.html.

[35] Inner Mongolia Autonomous Region Land and Resources Department Notice on the Public Selection and Evaluation Agency to Undertake Three Mining Rights Assessment Projects Such as the Survey and Exploration Rights of the Amanwusu Copper-Molybdenum Mine in Sunitezuo Banner, Inner Mongolia. http://zrzy.nmg.gov.cn/zwgk/tztg/201206/t20120613_29020.html.

[36] Institute of Hydrogeology and Environmental Geology, Chinese Academy of Geological Science (2011). Geological Environmental Protection and Treatment Restoration Plan for Shengli No. 1 Open-pit Mine of China Shenhua Energy Co., Ltd..

[37] West Ujimqin banner People’s Governmenet of Xilingol, Inner Mongolia. Baiyinhua Coal and Electricity Co., Ltd. http://www.xwq.gov.cn/tzxw/qyml/201312/t20131225_1155059.html.

[38] West Ujimqin Banner People’s Governmenet of Xilingol, Inner Mongolia Project Introduction of Baiyinhua No. 3 Surface Mine. http://www.xwq.gov.cn/tzxw/qyml/201312/t20131225_1155066.html.

[39] Ma S. (2011). Ecological Risk Assessment of Vulnerable Mine Area-Taking Shengli East No.2 Open-Pit as An Example. Master’s Thesis.

[40] Wu H.Y. (2014). Optimization of Mining Procedure in Baiyinhua No.1 Open Coal Mine. Master’s Thesis.

[41] Liu R.J. (2014). Study on Open-Pit Mine Safety Evaluation System and Evaluation Method for Baiyinhua No.4. Master’s Thesis.

[42] Inner Mongolia Haizhou Open-Pit Energy Co., Ltd. The first public announcement of the environmental impact assessment of the second phase of the Baiyinhua No. 4 phase Ⅱ (underground mine) of Inner Mongolia Haizhou Open-pit Energy Co., Ltd. http://www.xwq.gov.cn/xwq_info/qzf/zfxxgksx/tzgg/201701/t20170109_1705218.html.

[43] Tiandi Science & Technology Co., Ltd. (2006). Duolun Coal Mine Organization Construction Design.

[44] Bao J.J. (2004). The Researching of Environmental Assessment of Huolinhe South Open Pit Coal Mine. Master’s Thesis.

[45] Chinese Power Investment Mengdong Energy Group Co., Ltd. (2010). Zhahanao’er Open-Pit Coal Mine (Enlarged Area) Mining Right Evaluation Report of Chinese Power Investment Mengdong Energy Group Co., Ltd..

[46] Yang Y.C. (2014). Analysis of Mine Water Filling Factors and Research of Mine Water Prevention in Jinyuanli. Master’s Thesis.

[47] Wang Y. (2016). South-Eastern Slope Stability in Yuanbaoshan Surface Mine for Shift of Yingjinhe River. Master’s Thesis.

[48] Beijing Zhongtianhua Asset Appraisal Co., Ltd. (2006). Fengshuigou Coal Mining Right Evaluation Report of Inner Mongolia Pingzhuang Coal Industry (Group) Co., Ltd..

[49] Beijing Zhongtianhua Asset Appraisal Co., Ltd. (2013). Laogongyingzi Coal Mining Right Evaluation Report of Inner Mongolia Pingzhuang Coal Industry (Group) Co., Ltd..

[50] Beijing Zhongtianhua Asset Appraisal Co., Ltd. (2006). Liujia Coal Mining Right Evaluation Report of Inner Mongolia Pingzhuang Coal Industry (Group) Co., Ltd..

[51] Zhuge W.Y., Yue Y.J., Shang Y.R. (2019). Spatial-Temporal Pattern of Human-Induced Land Degradation in Northern China in the Past 3 Decades—RESTREND Approach. Int. J. Environ. Res. Public Health.

[52] Yan S.S., Li J., Yang Z. (2018). Dynamicremote sensing monitoring on the temporal-spatial changes of vegetation coverage in Chen Barag Banner from 2000 to 2016. J. China Agric. Univ..

[53] Chen X.Q., Wang H. (2009). Spatial and Temporal Variations of Vegetation Belts and Vegetation Cover Degrees in Inner Mongolia from 1982 to 2003. Acta Geogr. Sin..

[54] Mu S.J., Li J.L., Chen Y.Z., Gang C.C., Zhou W., Ju W.M. (2012). Spatial Differences of Variations of Vegetation Coverage in Inner Mongolia during 2001-2010. Acta Geogr. Sin..

[55] Zhang Y.F., Liang W.T., Liao Z.L., Han Z.H., Xu X.M., Jiao R., Liu H.L. (2019). Effects of climate change on lake area and vegetation cover over the past 55 years in Northeast Inner Mongolia grassland, China. Ttheor. Appl. Climatol..

[56] Zhuo Y., Yu F.M., Bao Y.H. (2007). Remote Sensing Monitor of the Ecological Environment of the Yimin Open Coal Mine in Inner Mongolia. J. Inner Mong. Norm. Univ. Nat. Sci. Ed..

[57] Li Y. (2016). The variation characteristics of temperature in Tongliao for Nearly 60 years and impacting on agricultural production. J. North. Agric..

[58] Wu W.Q., Li C.Y., Guo Y., Tong S.R., Han C., Liu J. (2007). Analysis of Temperature and Precipitation in Tongliao City in Recent 100 Years. Meteorol. J. Inner Mong..

[59] Zhang X.W., Wang J., Gao Y., Wu L.D., Ding Y.L., Bao C. (2018). Responses of Vegetation Changes to Climatic Variations in Hulunbuir Sandy Grassland in the past 15 Years. Acta Agrestia Sin..

[60] Hang Y.L., Bao G., Bao Y.H. (2014). Burenjirigala., Altantuya Dorjsuren. Spatiotemporal Changes of Vegetation Coverage in Xilin Gol Grassland and Its Responses to Climate Change during 2000-2010. Acta Agrestia Sin..

[61] Shao Y.P. (2017). Study on Dynamic Monitoring and Vegetation Coverage of Rare Earth Mines. Master’s Thesis.

[62] Shenhua Baorixile Enery Company Ltd. (2017). Report on Land Reclamation Plan for Reconstruction and Expansion Project of Inner Mongolia Baori Xierai Open-Pit Coal Mine.

[63] Shenhua Baorixile Enery Company Ltd. (2009). Open-Pit Coal Mine Mountain Environmental Protection and Comprehensive Treatment Plan.

[64] Liu Z.C., Han M. (2017). Initial effect and measures of slope treatment in Zhanihe Open-pit Mine. Coal Sci. Technol..

[65] Zhang Z.F. (2010). Application of Spraying Vegetation and Slope Protection Technology on Soil. Inner Mong. Water Resour..

[66] Rong Y., Hu Z.Q., Fu Y.H., Li D.M., Wan X.S., Anna W. (2017). Comparative study on land reclamation technology in typical surface coal mine in steppe region between China and USA. China Min. Mag..

